# Laparoscopic Management of Mechanical Small Bowel Obstruction Secondary to Meckel's Diverticulum with a Double Basis. A Rare Anatomic Presentation

**DOI:** 10.1055/s-0040-1713902

**Published:** 2020-11-23

**Authors:** Aurora Mariani, Mohammed Siddiqui, Nicolas Boulard, Dominique Berrebi, Arnaud Bonnard

**Affiliations:** 1 Department of Paediatric Surgery, Hopital Femme Mere Enfant, Bron, Auvergne-Rhône-Alpes, France; 2 Department of Pediatric Surgery, Robert-Debré Mother-Child University Hospital, Paris, Île-de-France, France; 3 Department of Pediatric Surgery, Hopital Jean Minjoz, Besancon, France; 4 Department of Pediatric Pathology, Robert-Debré Mother-Child University Hospital, Paris, Île-de-France, France; 5 Department of Pediatric Surgery and Urology, Robert-Debré University Hospital, Assistance-Publique Hôpitaux de Paris, Paris, France; 6 Sorbonne Paris Cité, Universite Paris Diderot, UMR 1149 Inserm, Paris, Île-de-France, France

**Keywords:** Meckel's diverticulum, minimal invasive surgery, laparoscopy, omphalomesenteric duct, intestinal obstruction

## Abstract

Meckel's diverticulum (MD) is the most common congenital abnormality of the gastrointestinal tract. Intestinal occlusion due to MD is a commonly observed consequence of intussusception or volvulus. Here, we report a case of an 11-year-old boy who presented to the emergency department with acute abdominal pain, bilious vomiting, and abdominal rebound tenderness. Computed tomography scan concluded a diagnosis of intestinal occlusion with no apparent cause. The patient was submitted to diagnostic laparoscopy, and mechanical occlusion by the permeable Meckel with double base was identified. A diverticulectomy by staplers was performed, and occlusion was alleviated. MD is a rare disease (for only 0.3–2.9% of the general population), and only 4.2 to 9% of patients diagnosed with MD have associated complications. MD can be a large base or a narrow base, with a mesodiverticular band but the diverticulum is usually a blind recess. In our case, the tube connected two intestinal segments. To the best of our knowledge, we have reported the first case of an MD-like permeable tube with a double basis.

## New Insights and the Importance for the Pediatric Surgeon

Meckel diverticulum should be considered in differential diagnosis in children presented with primary intestinal occlusion. Laparoscopic diverticulectomy is an accepted technique for the surgical treatment.

## Introduction

Meckel's diverticulum (MD) is a remnant of the embryologic vitelline (omphalomesenteric) duct that connects the fetal gut with the yolk sac and normally involutes between the 5th and 7th weeks of gestation. Failure or incomplete obliteration of vitelline duct results in certain congenital abnormalities. 1 In addition, some of these abnormalities may depend on the stage where the closure is stopped such as persistence of omphalomesenteric duct, MD, omphalomesenteric cyst, mesodiverticular bands, umbilical sinus, and umbilical polyp.

Among these anomalies, MD is the most frequently observed. 1 2 3 4 It is usually a blind recess on the mesenteric border of the terminal ileum, located 15- to 120-cm proximal to the ileocecal valve. A recent systematic review reported the prevalence between 0.3 and 2.9% in the general population and a life-time risk for symptoms at 4.2 to 9%. 5

Herein, we report a case of intestinal occlusion due to a permeable MD, consisting of a tubular structure with two bases (ends) connected to the ileum. To the best of our knowledge, it is the first such case to be reported in the literature.

## Case Report

An 11-year-old male child presented at the emergency department with acute abdominal pain and bilious vomiting. His physical examination revealed a distended abdomen with generalized rebound tenderness, and his rectal examination was unremarkable.

His blood test revealed an elevated leukocyte count (15,000/μL), normal renal function, and a normal hemoglobin level. An abdominal radiograph revealed dilated small bowel loops and computed tomography scan showed intestinal occlusion with no apparent cause. His small intestine was very dilated.

No other preoperative radiological investigations were performed. Initially, intravenous fluid resuscitation was provided to the patient, and subsequently, laparoscopy was performed.

**Fig. 1:**
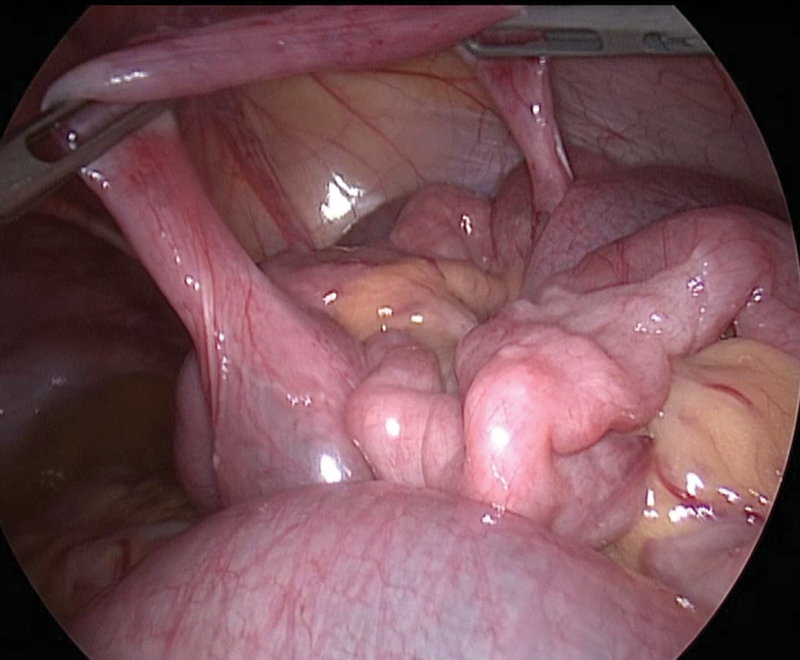
First view of Meckel's diverticulum. The structure is not inflammatory, with the same vascularization of the rest of the bowel.

**Fig. 2:**
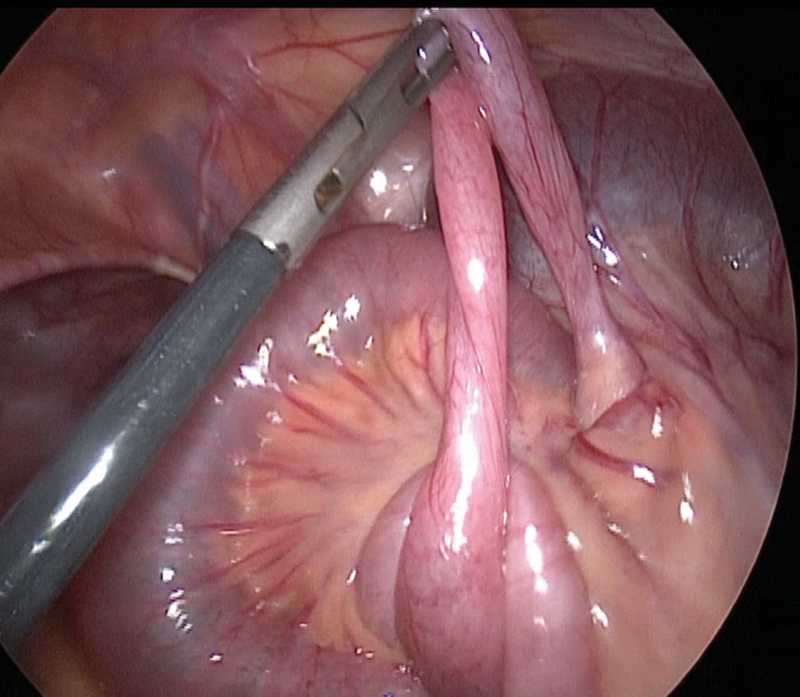
Laparoscopic view of MD with two bases. MD, Meckel's diverticulum.

In the laparoscopic approach, we used the open Hasson technique to insert a 10-mm port into the umbilicus. A 5-mm trocar was introduced in the suprapubic area, and another 5-mm trocar was introduced in the left iliac fossa under direct vision. On exploratory laparoscopy, the small bowel was dilated. We introduced another 5-mm trocar in the right hypochondrium to improve the exploration and found a tubular structure on the free border of the small bowel approximately 25 cm away from the ileocaecal valve. This tube was neither inflamed nor necrotic with the same vascularization of the rest of the bowel ( Fig. 1 ). Following this structure, we arrived at the other end that was attached to the small bowel on a free border and created a communication between these two parts of the bowel at 40 cm ( Fig. 2 ). The remaining portion of the intestine was incarcerated under the band.

**Fig. 3:**
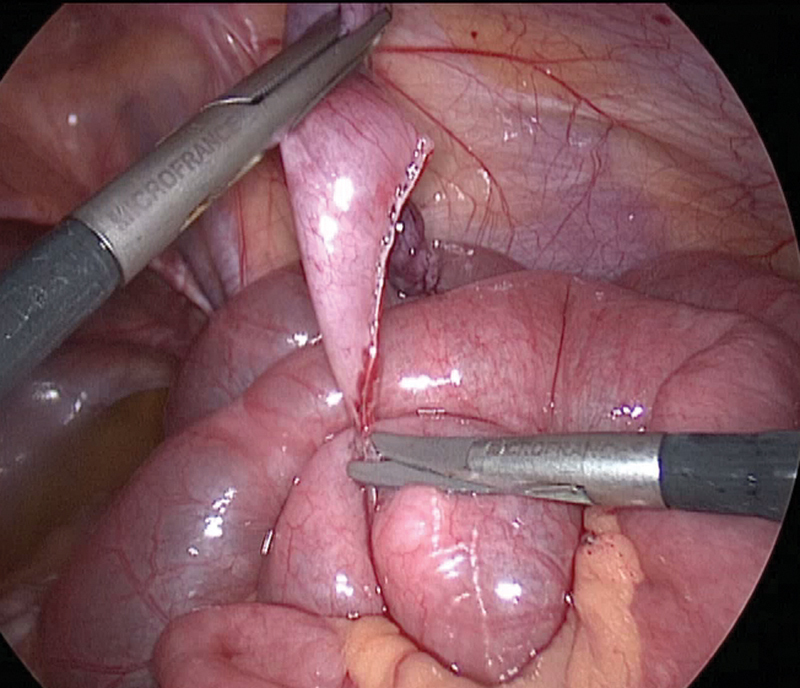
Laparoscopic stapler diverticulectomy. The first base.

**Fig. 4:**
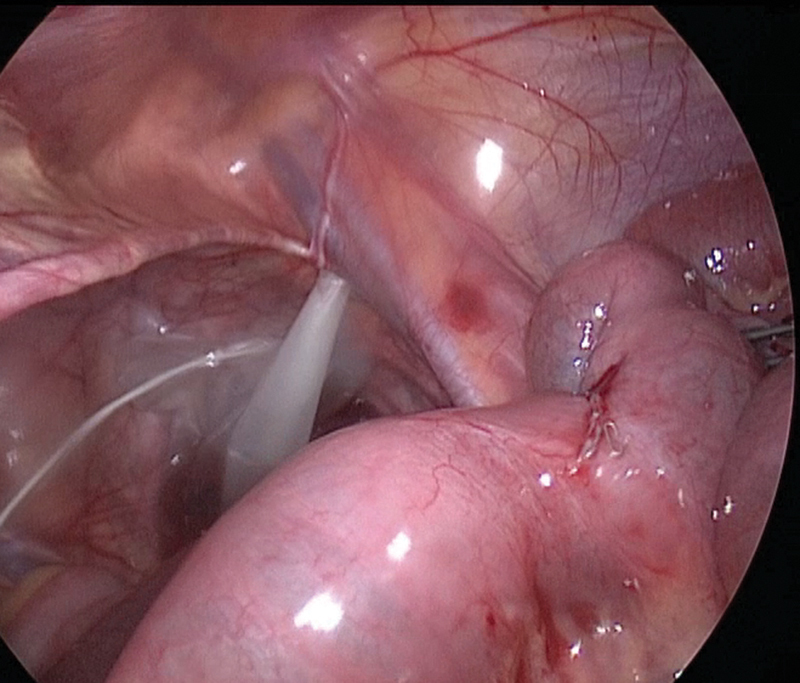
Bowel after MD resection with EndoGia. MD, Meckel's diverticulum.

There was a perforation due to important dilated loops of bowel above the zone of obstruction. Accordingly, it was decided to resect this diverticulum. A third trocar was inserted in the right flank area to help with this resection from its both ends with a 5-mm mechanical suturing EndoGia. It was applied to the two bases of the diverticulum, perpendicular to the bases but transverse to the longitudinal axis of the bowel ( Fig. 3 ). The stapler was fired, and the diverticulum was resected off the ileum ( Fig. 4 ). The punctiform and diastatic perforation were sutured intracorporeally with 3/0 Vicryl stitch. Rest of the bowel was examined, and no anomalies were observed. Peritoneal washing was done, and the specimens were delivered through umbilical port with the use of an Endobag. Abdominal fascia was closed with 2/0 Vicryl, and the skin was sutured with intradermic 5/0 Monocryl.

Examination of the specimen showed a hollow tubular structure with a diameter of 1.7 cm of both extremities with a mean tubal diameter of 1 cm. The structure was tubular all the way. The histopathology report revealed small bowel mucosa, gastric glands, and hemorrhagic tissue, indicating MD with gastric heterotopia.

The patient's recovery was uncomplicated, and he was discharged on the postoperative day 10.

## Discussion

MD is a persistent little portion of the vitelline duct, which arises from the antimesenteric border of the small intestine, located 40- to 60-cm proximal to the ileocecal valve. 5 In the embryo during intestinal rotation, the extremities of this vitelline duct can form a band containing a cyst in the middle (cyst vitelline). In other instances, the duct is permeable and hollow, reaching the umbilicus from the antimesenteric border of the ileum (fistula). 6 In 75% of cases, the tip of the diverticulum is usually unattached; however, in some cases (25%), it may attach to another organ or structure by means of a fibrous band. 7

In our case, the MD was seen as a double-based tube with both bases implanted on a part of the ileum permeable at both ends and hollow throughout the tubular cavity. It is difficult to describe this observation with classical embryogenic theories. We believe that after the reintegration of the primitive intestinal parts into the abdomen, the tip of the vitelline canal forms a vitelline fistula extending from distal ileum without closure of its distal extremity like in a classical MD. In our case, the tip of MD is the fistula, attached to an intestinal segment, and located 40-cm proximal to the ileocaecal valve. An old perforation of this MD with secondary fistulation is improbable due to the total integrity of the resected tubal structure, with no peritoneal adherences and lack of any symptoms before this episode. To the best of our knowledge, this is the first case describing a diverticulum connecting two loops of bowel.

In our case, the MD was like a band that formed a loop, through which loops of small bowel became entrapped and incarcerated. Currently, laparoscopy is an accepted technique for establishing the diagnosis and subsequent management of MD in children. 8 9 Various studies have demonstrated that diverticulectomy alone was sufficient to clear all heterotopic tissues without increasing the risk of continued bleeding or other complications compared with segmental small bowel resection. Moreover, diverticulectomy appears to have a lower overall complication rate. 10 11 Diverticulectomy can be performed either intracorporeally or extracorporeally. Intracorporeal diverticulectomy can be performed laparoscopically without the need to enlarge the umbilical wound. Endoscopic staplers were the most frequently reported device used in this surgery.

According to the match we had between the clinical presentation and the literature, we did a direct resection by a mechanical EndoGia stapler at both ends at their bases where they were implanted into the small bowel. The vision we had during the procedure under the laparoscopy was very satisfactory.

## Conclusion

To the best of our knowledge, we are the first to report an MD-like tube with double bases. Based on our findings, we can infer that laparoscopic surgical management was an adequate choice in our case.
